# Development of a dental handpiece angle correction device

**DOI:** 10.1186/s12938-018-0606-1

**Published:** 2018-11-26

**Authors:** Yoon Nam, Mi Young Eo, Soung Min Kim

**Affiliations:** 1 Department of Oral Medicine and Oral Diagnosis, School of Dentistry, Dental Research Institute, Seoul National University, Seoul, South Korea; 2 Department of Oral and Maxillofacial Surgery, School of Dentistry, Dental Research Institute, Seoul National University, 101 Daehak-ro, Jongno-gu, Seoul, 110-768 South Korea

**Keywords:** Angulation correction device, Convergence angle, Tooth axis deviation, Gyro sensor

## Abstract

**Background:**

Preparation of a uniform angle of walls is essential for making an ideal convergence angle in fixed prosthodontics. We developed a de novo detachable angle-correction apparatus for dental handpiece drills that could help the ideal tooth preparation.

**Methods:**

We utilized a gyro sensor to measure the angular velocities to calculate the slope of an object by integrating the values, acceleration sensor to calculate the slope of an object by measuring the acceleration relative to gravity, and Kalman filter algorithm. Converting the angulation of the handpiece body to its drill part could be performed by a specific matrix formulation set on two reference points (2° and 6°). A flexible printed circuit board was used to minimize the size of the device. For convergence angle investigation, 16 volunteers were divided randomly into two groups for performing tooth preparation on a mandibular first molar resin tooth. All abutments were scanned by a 3D scanner (D700^®^, 3Shape Co., Japan), the convergence angle and tooth axis deviation were analyzed by a CAD program (SolidWorks 2013^®^, Dassault Systems Co., USA) with statistical analysis by Wilcoxon signed-rank test (α = 0.05) using SPSS statistical software (Version 16.0, SPSS Inc.).

**Results:**

This device successfully maintained the stable zero point (less than 1° deviation) at different angles (0°, 30°, 60°, 80°) for the first 30 min. In single tooth preparation, without this apparatus, the average bucco-lingual convergence angle was 20.26° (SD 7.85), and the average mesio–distal (MD) convergence angle was 17.88° (SD 7.64). However, the use of this apparatus improved the average BL convergence angle to 13.21° (SD 4.77) and the average MD convergence angle to 10.79° (SD 4.48). The angle correction device showed a statistically significant effect on reducing the convergence angle of both directions regardless of the order of the directions.

**Conclusions:**

The angle correction device developed in this study is capable of guiding practitioners with high accuracy comparable to that of commercial navigation surgery. The volume of the angle correction device is much smaller than that of any other commercial navigation surgery system. This device is expected to be widely utilized in various fields of orofacial surgery.

## Background

Today, the needs for precise dental procedures have increased. In tooth preparation and implant placement, which are the two main axes of modern dental procedures, there have been a myriad of developments to meet those needs. Accuracy not only improves the quality of the dental procedures, but also reduces the time required, allowing for minimally invasive techniques to reduce postoperative complications [1]. The accuracy of angulation is especially emphasized for tooth preparation of full-crowns and placement of implant fixtures [2, 3]. During patient functioning, in order to keep the fixed prosthesis stable on the tooth, proper resistance and maintenance of the abutment form are necessary. The maintenance of cast prosthesis is determined by convergence angle of the abutment, contact area, inner surface roughness of the structure, etc. [4, 5]. Among them, convergence angle is considered a primary factor and has been the main focus of most studies [6]. The definition of convergence angle is ‘the angle between opposing axial walls and has been shown to affect crown retention’. It has also been determined that the optimal convergence angle ranges from 5 to 12° [7, 8].

The accuracy of angulation in dental procedures is important for minimizing adverse effects, ensuring ideal aesthetic results, and maintaining proper oral health. Ideally, the implant should be placed parallel to other adjacent implants or remaining teeth. This results in proper application of vertical occlusal forces to the implant. In particular, when more than one dental implant has been placed, the angle between them affects the maintenance of the upper prosthesis. A study on the relationship between retention of the prosthesis and inter-implant angle in 24 overdenture-wearing patients reported that, as inter-implant angle was increased, retention of the implant overdenture using locator attachments significantly decreased [9]. In addition, when using the angled abutment to compensate for angulation error, the stress on the implant and surrounding bone has been found to increase. However, the increased stress did not exceed physiological limits [10]. The ability to create the correct angulation is an important dental technique. However, there must be a discrepancy between reality and theory when performing this technique with a dental handpiece. This discrepancy is well-described in the studies about convergence angles for abutments for casting crowns. Analysis of the convergence angle of 478 clinically formed abutments reported that the actual average convergence angle was 21°, with a large variance among the dentists [11]. Other studies also supported that finding by reporting the average convergence angle ranging from 14 to 20°, which is considerably deviated from the ideal value [6, 12]. An increase of convergence angle results in a reduction of the average retention force regardless of the type of cement used [13]. Implant placement procedures have already reached a level of computer guidance that implements preoperative planning, surgical guiding, and uses an optical tracking device [4, 14, 15]. In addition, there is a method using an electromagnetic tracker and a direct surveying apparatus such as a Parallel-A-Prep [16, 17].

In this paper, a de novo detachable dental angulation correction device is proposed to overcome the disadvantages of conventional guiding devices and to achieve the requirements listed above.

## Methods

### Principle of angulation measurement

We used the MPU-6050^®^ module containing a gyro sensor and acceleration sensor (TDK InvenSense^®^, Mouser Electronics Co., USA) commercialized in the field of mobile devices and drones to measure the three-dimensional angulation of the dental drill. The gyro and acceleration sensors measure the angular velocity and relative acceleration of gravity, respectively. When integrating the angular velocity measured from the gyro sensor, the angular deviation can be obtained from the original state. When using the acceleration sensor, it is possible to measure the extent of the device tilted from the original state by measuring acceleration relative to gravitational acceleration. Both sensors have complementary pros and cons. Although the stabilized value of the gyro sensor is sensitive to a single rotation, its reliability decreases over time because of the zero point shift. On the contrary, the acceleration sensor exhibits no zero point drift with the lapse of time, but its accuracy is variable due to translational movement. To compensate for the errors caused by these technical limitations, several algorithms such as the Kalman filter have been applied [18]. For the device developed in this study, gyro and acceleration sensors are simultaneously utilized to calculate the angle of the drill while applying the Kalman filter algorithm.

### Computation and display process

The computational process of the device can be divided into three phases. The first phase involves setting the reference angulation. This process begins by contacting the tip of drill to the tooth surface. At the contact point, the practitioner presses the pedal with her/his free foot to retain the spatial angle of the sensor and use that angle as the reference. After noting the reference angle, the second phase involves translation of the three axial angles of the handpiece body to the dental drill. Almost every commercially available handpiece has a drill-containing head part that is not parallel to the hand-holding body part.

**Fig. 1:**
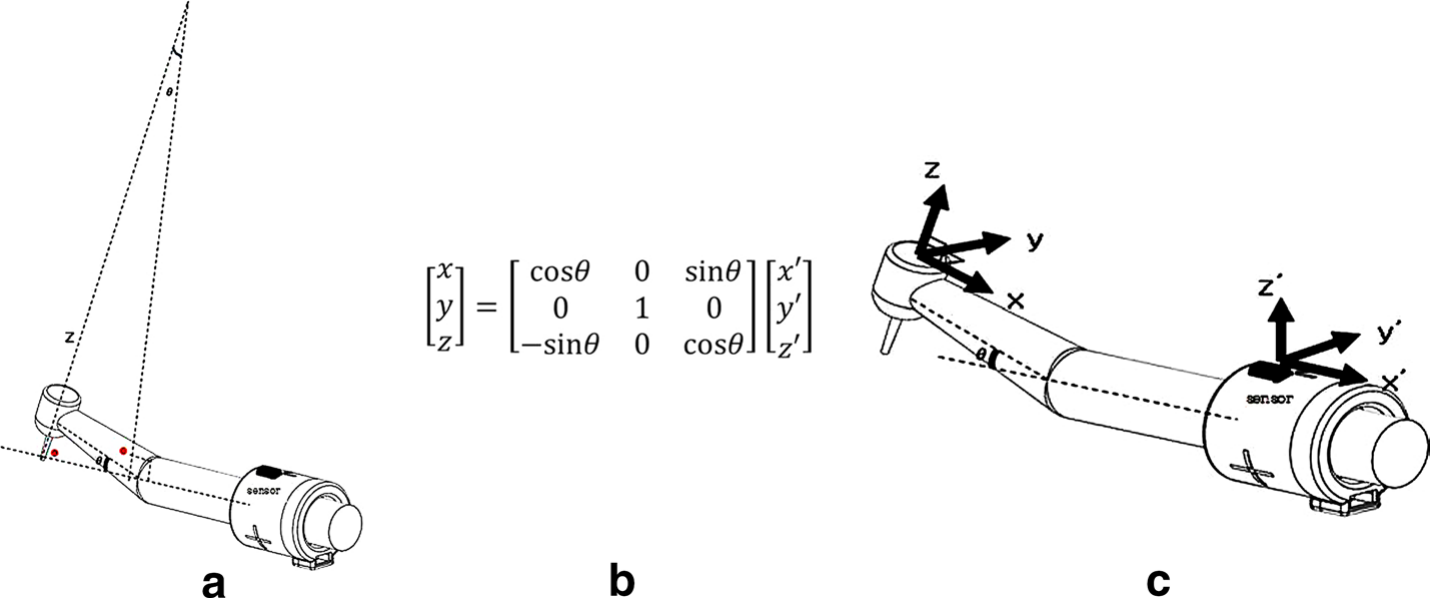
Angled configuration of the designed handpiece in this study. When the angle between the head and body portion of the hand piece is “θ,” the angle between a line perpendicular to the sensor and a line extended from the axis of the drill is also “θ” (**a**). The body to head transition matrix formula (**b**) translates the three axial angles of the hand piece body (x’, y’, z’) into those of the dental drill as x, y, and z, respectively (**c**). Due to this formula, we do not have to create a different electric circuit board for every different type of handpiece. Since it is the dental drill that reduces the tooth structure and not the handpiece, this process improves the clinical efficacy of the device

When the angle between the head and body portion of the hand piece is “θ,” the angle between a line perpendicular to the sensor and a line extended from the axis of the drill is also “θ” (Fig. 1a). Therefore, as depicted in Fig. 1b, c, a specific formula translates the three axial angles of the handpiece body (x’, y’, z’) into those of the dental drill (x, y, z). In addition, this process increases the clinical efficacy of the device because it is the dental drill that removes tooth structure, not the handpiece. The third phase tracks the change in angulation of the three axes caused by the rotation of the handpiece (from x_1_, y_1_, z_1_ to x_2_, y_2_, z_2_). Since rotation of the dental drill along the z-axis does not change the clinical spatial angle, this device only displays the angulation for x- and y-axes. Using this bypass, clinicians can exclude the error caused by simple positional change of the hand and body. In addition, this device uses two reference points. The first reference point is 2°, which is set by the user. LED indicators for each axis flash when the dental drill is out of the tolerable angular range (2° for each reverse axis). If the handpiece drill is located in the first reference position, the LED indicators are automatically switched off.

The clinician is able to maintain the original angulation when he/she tilts the drill according to the flashing LED indicators. For instance, when the drill is tilted toward the + x direction by 3°, the LED indicator of − x will blink. If the clinician adjusts the angulation by tilting the drill toward the − x direction, the LED indicator for − x will be turned off. The indicators for the x and y axes of the dental drill operate independently. The second reference point is 6°, which is set by the user. The LED indicator for each axis is constantly lit when the dental drill is moved out of the second reference angle (by 6°) for each reverse axis. For example, when the drill is tilted toward the + x direction by 10°, the LED indicator of − x will be turned on. If the clinician adjusts the angulation of the dental drill to the proper position, the indicator will be turned off or blink.

### Use of a flexible electronic circuit board (fPCB)

**Fig. 2:**
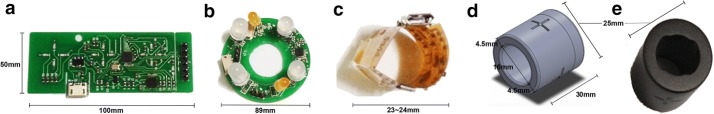
An experimental electric circuit (**a**) was tested on a bread board (**a**), and then this circuit was printed on a round printed circuit board (PCB) (**b**). This flexible PCB could be rolled into a round form due to its bending property (**c**); as a result, the diameter of the PCB was reduced to 25 to 26 mm for adaptation into the dental handpiece. A 3-dimensional model (**d**) with a prototype (**e**) was created according to the spatial coordination of the circuit board

This device utilizes a flexible PCB to dramatically reduce the size and thickness of the device and improve clinical efficacy (Fig. 2). To comfortably grab this device with the index finger and thumb, the maximum diameter of the apparatus should be less than 26 mm.

### Effectiveness and efficacy of the device

#### Zero point drift

To evaluate the zero point drift of the device, we measured the x angulation at various angles (0°, 30°, 60°, 80°, and 90°) when the device was in a stationary state. Data was collected for 30 min. According to in vitro studies, the minimum security distance was recommended at 1 mm [19–21]. The ‘30 min’ criterion was established to allow sufficient time for each dental practice. Therefore, in this study, we defined the stabilized zero point as ‘less than 1 mm deviation for 30 min’.

#### Reduction of the convergence angle during single crown preparation

Sixteen volunteers were recruited to participate in the study. Volunteers were composed of 14 dental students (3rd and 4th years in Seoul National University School of Dentistry) and two prosthetics residents. They were divided randomly into 2 groups (1 and 2). The only difference between these groups was the timing of device use. A dental simulator (Nissim type 1^®^, Nissan Co., Japan), Dentiform (PRO2002-UL-SP-FEM-28^®^, Nissan Co., Japan), and #36 tooth model (A5A-200^®^, Nissan Co., Japan) were used.

When the volunteers used the apparatus, they created a 1.5-mm-deep punch out (at the reference angulation), which was parallel to the preferred tooth axis at the center of the occlusal plane. According to the instruction of the apparatus, the volunteers performed tooth preparation within the range of 2 to 6° of deviation from the reference angulation. When the volunteers did not use the apparatus, they performed usual tooth preparation for a full veneer gold crown without marking the reference angulation.

All abutments were scanned by a 3D scanner (D700^®^, 3Shape Co., Japan). The convergence angles were analyzed by a CAD program (SolidWorks 2013^®^, Dassault Systems Co., USA). The midline of each axial wall was selected to measure the convergence angle. Data was statistically analyzed by Wilcoxon signed-rank test (α = 0.05) using SPSS statistical software (Version 16.0, SPSS Inc.).

#### Reduction of convergence angle during 3-unit bridge crown preparation

Sixteen volunteers were years in Seoul National University School of Dentistry) and two prosthetics residents. They were randomly divided into 2 groups (1 and 2). The only difference between these two groups was the timing of device use. A dental simulator (Nissim type 1^®^, Nissan Co., Japan), Dentiform (PRO2002-UL-SP-FEM-28^®^, Nissan Co., Japan), #36 tooth mode l (A5A-200^®^, Nissan Co., Japan), and #34 ideal abutment mode l (A21A-LL42^®^, Nissan Co., Japan) were used to mimic real clinical situations.

**Fig. 3:**
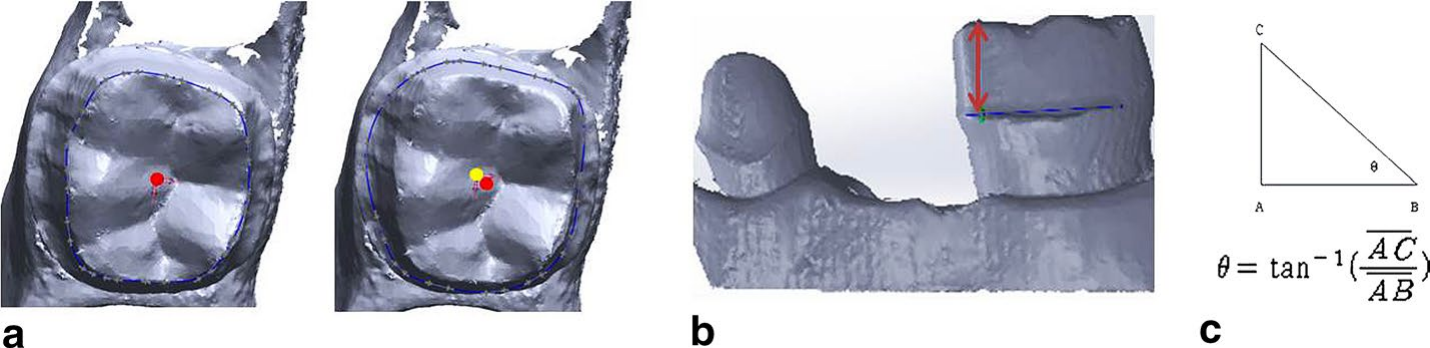
Calculation of tooth axis (**a**), tooth height (**b**), and tooth axis angulation (**c**). When observed from a fixed view point, the line between the centers of gravity at the top (red spot) and bottom (yellow spot) surfaces was defined as the tooth axis of the abutment. When the center of gravity of the top and bottom surfaces is (x_t_, y_t_, z_t_) and (x_b_, y_b_, z_b_), respectively, the tooth axis is $\overrightarrow {Axis} = \left( {x_{t} - x_{b} ,\,y_{t} - x_{b} ,\,z_{t} - z_{t} } \right)$ (**a**). The height of the abutment was defined as the distance from the upper abutment margin to the bucco-mesial cusp tip (**b**). Tooth axis angulation was obtained for each direction using the inverse tangent function (**c**)

The axial walls of the #34 ideal abutment teeth were used as a reference. When the volunteers used the apparatus according its instructions, they performed tooth preparation within the range of 6° of deviation from the reference angulation. When the volunteers did not use the apparatus, they performed usual tooth preparation for a 3-unit bridge prosthesis without setting a reference point. All abutments were scanned by a 3D scanner (D700^®^, 3Shape Co., Japan). The convergence angles were analyzed by a CAD program (SolidWorks 2013^®^, Dassault Systems Co., USA). When observed from a fixed point of view, the line between the centers of gravity at the top and bottom surfaces was defined as the tooth axis. By measuring the discrepancy between those two points and the height of the abutment, we obtained the tooth axis angulation for each direction (Fig. 3). To analyze tooth axis deviation, we measured the angle between tooth axes of the #34 and #36 abutments. Data was statistically analyzed by Wilcoxon signed-rank test (α = 0.05) using SPSS statistical software (Version 16.0, SPSS Inc.).

## Results

### Novel angle correction device

**Fig. 4:**
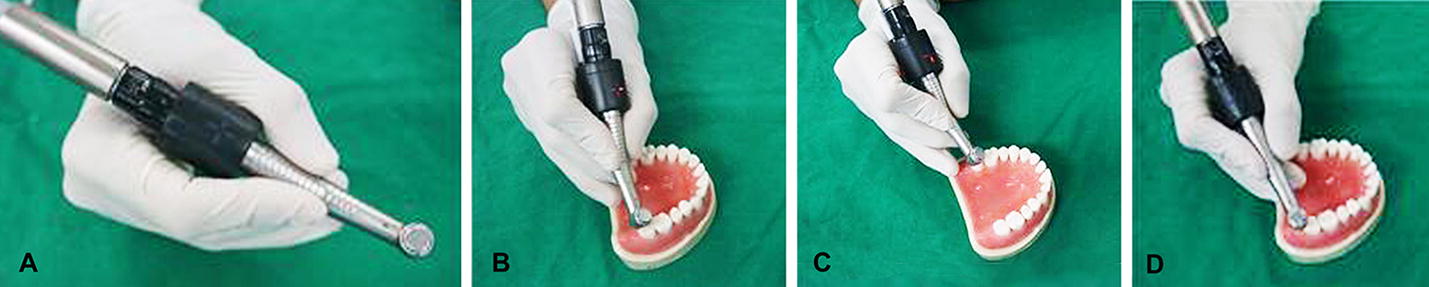
The operative appearances of the angle correction device mounted to the dental handpiece (**A**), − x deviation of the handpiece from the reference showing the red LED indicator (**B**), − y deviation of the handpiece from the reference showing the red LED indicator (**C**), and a regression to the reference showing all LED indicators turned off (**D**)

The maximum diameter was 26 mm. Figure 4 shows the appearance and operation of the device (Fig. 4). There were two reference points: 2° (blinking) and 6° (constantly lit). When the angulation of the drill deviates from the reference for 2°, the LED indicator of the opposite direction (same axis) blinks. The practitioner is then prompted to tilt the handpiece drill toward the direction in which the LED indicator blinks. The LED indicators of different axes operate independently. Since the z-axis is parallel to the drill, one can ignore any associated rotation. Therefore, despite the angle of adjustment in 3-dimensional space, there are only 2 directions to adjust.

### Zero point drift

**Fig. 5:**
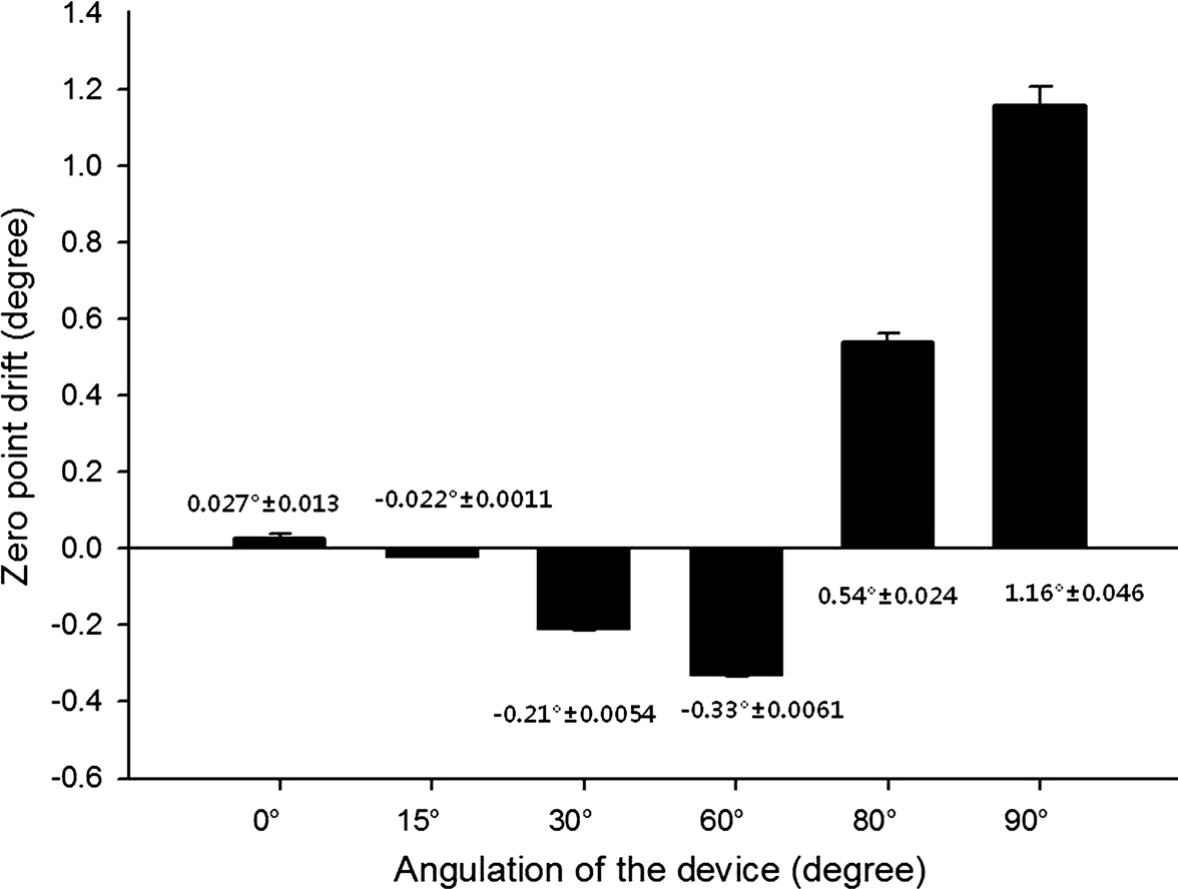
Zero point drift after 30 min at various angles showing that the zero point drift was less than 1° at the five angles of 0°, 15°, 30°, 60°, and 80°. The 1° deviation criteria for the angle correction device is recommended from previous in vitro studies, but the average zero point drift exceeded the 1° of deviation (1.16° at 90°)

For the first 30 min, a de novo angle correction device successfully maintained the stable zero point (less than 1° of deviation) at four different angles (0°, 30°, 60°, and 80°). We measured the angle of the 3 axes every 0.1 s. According to previous in vitro studies, the minimum distance recommended for an implant surgical guide to prevent damage of anatomical structures is 1 mm [19–21]. For wider clinical applications, we used the same criterion for our device. Figure 5 shows average zero point movements after 30 min. However, after 30 min, the zero point changed by 1.16° from 90° (SD 0.046). Figure 5 shows the average zero point drift after 30 min (Table 1).

**Table 1:** Zero point drift at various angles

	0°*	15°	30°	60°	80°	90°
Drift (°)	0.027	− 0.022	− 0.21	− 0.33	0.54	1.16
Standard deviation	0.013	0.0011	0.0054	0.0061	0.024	0.046

* Average zero point drift on the x axis after 30 min

### Convergence angle

**Fig. 6:**
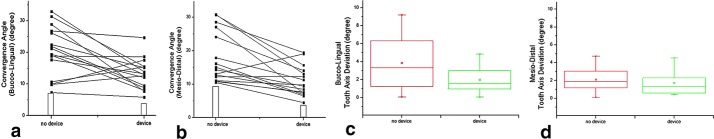
The effect of the angle correction device on convergence angle for bucco-lingual (**a**) and mesio–distal directions (**b**) and on tooth axis deviation for bucco-lingual (**c**) and mesio–distal directions (**d**)

When not using the apparatus, the average bucco-lingual convergence angle was 20.26° (SD 7.85), and the average mesio–distal convergence angle was 17.88° (SD 7.64). When using the apparatus, the average bucco-lingual convergence angle was 13.21° (SD 4.77), and the average mesio–distal convergence angle was 10.79° (SD 4.48). The angle correction device exhibited a statistically significant effect on reducing the convergence angle for both directions regardless of the order of direction (*p* = 0.023, 0.009 for bucco-lingual and mesio–distal, respectively) (Fig. 6a, b) (Table 2).

**Table 2:** The effect of correcting device on convergence angle

	Average	Standard deviation	p-value
(A) Convergence angle in the bucco-lingual direction			
No device	20.26°	7.85	0.023
Device	13.21°	4.77	
(B) Convergence angle in the mesio–distal direction			
No device	17.88°	7.64	0.009
Device	10.79°	4.48	

### Tooth axis deviation between the 3-unit bridge abutments

When not using the apparatus, the average bucco-lingual tooth axis deviation was 3.86° (SD 2.99), and the average mesio–distal convergence angle was 2.12° (SD 1.32). When using the device, the average bucco-lingual convergence angle was 2.00° (SD 1.47), and the average mesio–distal convergence angle was 1.71° (SD 1.33). The angulation correction device had a statistically significant effect on reducing bucco-lingual tooth axis deviation regardless of the order of using the device (*p* = 0.021). Although there was a reduction of tooth axis deviation on the mesio–distal side, it was not statistically significant (Fig. 6c, d) (Table 3).

**Table 3:** The effects of correcting device on tooth axis deviation

	Average	Standard deviation	p-value
(A) Tooth axis deviation in the mesio–distal direction			
No device	3.86°	2.99	0.021
Device	2.00°	1.47	
(B) Tooth axis deviation in the mesio–distal direction			
No device	2.12°	1.32	0.081
Device	1.71°	1.33	

## Discussion

In this study, a cylinder-shaped hollow detachable angle correction device was developed and tested on tooth preparations for a full veneer and a 3 unit-bridge prosthesis. Recently, several attempts utilizing gyro and acceleration sensors on dental handpieces have been made [22, 23]. However, those projects were only conceptual and therefore not commercialized because of numerous technical and clinical hurdles. Another main purpose of this study is to minimize inevitable human errors in various types of tooth preparations with sensor-mounted devices. In this aspect, this study is expected to have potential in improvement of technical accuracy in various types of dental practice. The convergence angle of the abutment largely affects the retention of restorative materials. Convergence angle can be defined as “the angle between opposing axial walls” and has been shown to affect crown retention. In addition, it has been demonstrated that the increase of convergence angle reduces the retentive force regardless of the type of cement [7, 13].

**Fig. 7:**
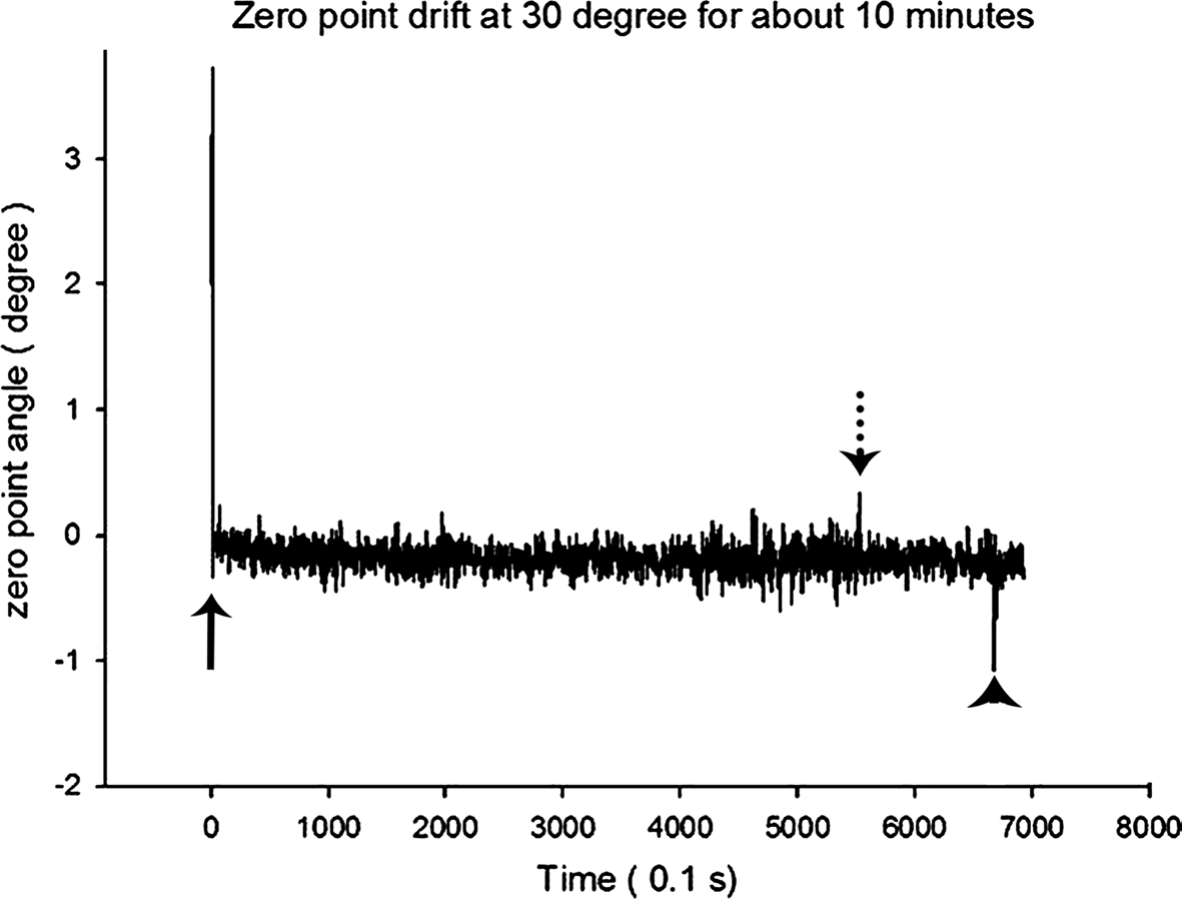
The data of the zero point angle of the x-axis at 30° for 10 min, showing the drift of the zero point during 699 s at 30°. The three arrows of full line, dotted line, and arrow head indicate the outranged values. The full line arrow shows the ‘zero point adjustment process’ that always occurs at the beginning of device operation for less than 1 s. There were other outranged values for the moment of 0.1 s shown with the dotted line arrow and arrow head. However, these outranged values were considered spontaneous noise because they did not induce any problems with maintaining the zero point value

Using a gyro sensor, the angular velocity was recorded and was integrated to measure the rotational angle. In conventional devices using this mechanism, the accumulated error causes drift of the zero point. In contrast, the acceleration sensor detects the relative acceleration corresponding with gravitational acceleration and thus minimizes the drift of the zero point. However, problems still exist with obtaining the slope since its accuracy is influenced by external forces and translational movements. To compensate for these errors, filtering algorithms such as the Kalman filter algorithm and compensatory filter algorithm were developed [18]. In this study, the zero point shift at various angles (0°, 30°, 60°, 80°, and 90°) was observed. To mimic the clinical condition, all the data were collected for 30 min. Zero point shift less than 1°, demonstrated as the minimum error for implant guide surgeries in previous studies, was set as the standard [19–21]. The result showed that there was no zero point shift observed except when the device was placed at 90° for 30 min. However, this shift was clinically negligible because maintaining the handpiece in a perpendicular position for 30 min without any movement is unreasonable and not likely to occur. The zero point shift measured in this study involved time-dependent average values, but the raw data showed an outranged value that deviated from the zero point for a moment (0.1 s) (Fig. 7). However, this outranged value can be considered spontaneous noise. Therefore, it can be stated that there was no problem maintaining the zero point value.

The practitioner group using the device developed in this study exhibited significantly decreased convergence angles (MD *p* = 0.009, BL *p* = 0.023). For the same practitioner group, the conventional handpiece resulted in convergence angle values of MD 17.88° and BL 20.26°, close to the values from previous studies of 21° and 14–20° [6, 12]. Another advantage of using this device is improved consistency. The distribution of the data collected in each trial decreased from MD 7.64° and BL 7.85° to MD 4.48° and BL 4.77°, respectively. For the #36 single crown full veneer preparation, there was a significant decrease in both average convergence angle and distribution at bucco-lingual and mesio–distal sides. This suggests that the device actually improves not only the accuracy, but also the precision of the practitioner’s work, allowing more credible results.

In preparation for a 3-unit bridge prosthesis, the axis of #34 tooth was set as the standard for the preparation of #36 tooth. After the preparations, the angle between the axes of each abutment was measured. For the bucco-lingual side, the average angle between the two axes decreased from 3.86° (SD 2.99) to 2.12° (SD 1.32) with statistical significance (*p *= 0.021). In preparation for the 3-unit bridge prosthesis, the angle between the two axes decreased from 2.00° (SD 1.47) to 1.71° (SD 1.33) at the mesio–distal side, but this change was not statistically significant (*p* = 0.081). Two possible explanations are discussed here; first, it is easier for the practitioner to perform accurate surgical procedures on the mesio–distal side versus the bucco-lingual side. Based on this result, it is possible to deduce that surgical procedures on the mesio–distal side require less technical skill than those on bucco-lingual side, and this difference is due to the spatial arrangement of the teeth. Therefore, improvement of clinical efficacy by using this device is less dramatic on the mesio–distal side. Secondly, since the mesio–distal length of the mandibular molar is longer than its bucco-lingual length, the same 0.5–1 mm preparation of different sides of the tooth might have resulted in a smaller amount of axial reduction on the mesio–distal side. As a result of the smaller amount of axial reduction, the smaller difference in angles between the two axes becomes not statistically significant.

The angle data collected from this device can be integrated and displayed to improve the practitioner’s accuracy in his/her dental procedures. The clinical efficacy of this device was tested in a dental simulator for the consideration of various clinical aspects. However, the data collected from the test cannot perfectly reflect real clinical situations because the patient’s head and mandible are not fixed as they are in the dental simulator. Therefore, the next generation of this device should be attachable to the relatively fixed anatomical structure of the patient. In addition, considering that angle detection by gyro and acceleration sensors is influenced by external forces such as tremors, use of vibration-proof materials for low speed handpieces or implant drills is highly recommended.

## Conclusions

We developed a de novo detachable angle-correction apparatus for the dental handpiece drill for minimized volume and improved convenience. This angle correction device is capable of guiding practitioners with high accuracy comparable to that of commercial navigation surgery. This device can be utilized in various fields of dentistry such as tooth preparation, implant placement, orthodontics, esthetics surgery, and education for novice dental students.
